# The status of emergency obstetric and newborn care in post-conflict eastern DRC: a facility-level cross-sectional study

**DOI:** 10.1186/s13031-021-00395-0

**Published:** 2021-08-11

**Authors:** Serge-André Mizerero, Calistus Wilunda, Patou Masika Musumari, Masako Ono-Kihara, Gerrye Mubungu, Masahiro Kihara, Takeo Nakayama

**Affiliations:** 1 Graduate School of Medicine, School of Public Health, Department of Health Informatics, Kyoto University, Kyoto, Japan; 2 African Population and Health Research Centre, Manga Close, P.O. Box 10787-00100, Nairobi, Kenya; 3 Interdisciplinary Unit for Global Health, Centre for the Promotion of Interdisciplinary Education and Research, Kyoto University, Yoshida honmachi, Sakyo-ku, Kyoto, 606-8501 Japan; 4 International Institute of Socio-Epidemiology, Kitagosho-cho, Sakyo-ku, Kyoto, 606-8336 Japan; 5 Department of Paediatrics, University Hospital of Kinshasa, School of Medicine, Kinshasa, Democratic Republic of the Congo

**Keywords:** Emergency obstetric and newborn care, Post conflict, Eastern Democratic Republic of the Congo, North-Kivu province, Process indicators, Maternal and newborn care

## Abstract

**Background:**

Pregnancy-related mortality remains persistently higher in post-conflict areas. Part of the blame lies with continued disruption to vital care provision, especially emergency obstetric and newborn care (EmONC). In such settings, assessment of EmONC is essential for data-driven interventions needed to reduce preventable maternal and neonatal mortality. In the North Kivu Province (NKP), the epicentre of armed conflict in eastern Democratic Republic of the Congo (DRC) between 2006 and 2013, the post-conflict status of EmONC is unknown. We assessed the availability, use, and quality of EmONC in 3 health zones (HZs) of the NKP to contribute to informed policy and programming in improving maternal and newborn health (MNH) in the region.

**Method:**

A cross-sectional survey of all 42 public facilities designated to provide EmONC in 3 purposively selected HZs in the NKP (Goma, Karisimbi, and Rutshuru) was conducted in 2017. Interviews, reviews of maternity ward records, and observations were used to assess the accessibility, use, and quality of EmONC against WHO standards.

**Results:**

Only three referral facilities (two faith-based facilities in Goma and the MSF-supported referral hospital of Rutshuru) met the criteria for comprehensive EmONC. None of the health centres qualified as basic EmONC, nor could they offer EmONC services 24 h, 7 days a week (24/7). The number of functioning EmONC per 500,000 population was 1.5. Assisted vaginal delivery was the least performed signal function, followed by parenteral administration of anticonvulsants, mainly due to policy restrictions and lack of demand. The 3 HZs fell short of WHO standards for the use and quality of EmONC. The met need for EmONC was very low and the direct obstetric case fatality rate exceeded the maximum acceptable level. However, the proportion the proportion of births by caesarean section in EmONC facilities was within acceptable range in the HZs of Goma and Rutshuru. Overall, the intrapartum and very early neonatal death rate was 1.5%.

**Conclusion:**

This study provides grounds for the development of coordinated and evidence-based programming, involving local and external stakeholders, as part of the post-conflict effort to address maternal and neonatal morbidity and mortality in the NKP. Particular attention to basic EmONC is required, focusing on strengthening human resources, equipment, supply chains, and referral capacity, on the one hand, and on tackling residual insecurity that might hinder 24/7 staff availability, on the other.

## Background

Emergency obstetric and newborn care (EmONC) is globally recognized as an essential health package for reducing preventable maternal and neonatal mortality, particularly in countries with persistently higher mortality rates [1–5]. Most of these countries are located in sub-Saharan Africa (SSA) [6, 7], a region that has witnessed the majority of armed conflicts over the past 3 decades [8]. Research shows that conflict disproportionately affects maternal and child health, both during and years after it has ended [9–15], and that many of the countries where most maternal and child deaths occur are experiencing or have emerged from conflict [16, 17]. Some have argued that reduced availability/low quality of EmONC services is ‘the single most important factor implicated in maternal deaths in conflict and post-conflict settings’(12). Accordingly, it is of vital importance to improve access to quality EmONC in conflict-affected settings through data-driven programming.

The EmONC life-saving services, or signal functions, define 2 types of complementary health facilities based on their capacity to provide, within a 3-month period, the 7 basic signal functions or all 9 signal functions when pregnancy- and childbirth-related complications occur (see Table 1). These complications, including haemorrhage, hypertensive disorders, sepsis, obstructed labour, complications of abortion, and intrapartum related asphyxia, cause most maternal deaths, stillbirths, and early neonatal deaths [18–20]. Their occurrence is often unpreventable [21], unforeseeable [1], and expected in about 15% of women during pregnancy, childbirth, and the immediate postpartum [22]. The disruptive and lingering effects of conflict on health services provision—deficient health personnel, damaged health infrastructure, inadequate healthcare coordination, and weak supply chains—contribute to increased vulnerability to adverse outcomes related to these complications [15, 23].

**Table 1:** Signal functions for basic and comprehensive EmONC health facilities

Basic EmONC	Comprehensive EmONC
#1. Administration of parenteral antibiotics	Performs #1 through #7, plus
#2. Administration of uterotonic drugs	#8. Surgery (Caesarean section)
#3. Administration of anticonvulsants	#9. Blood transfusion
#4. Manual removal of placenta (MRP)	
#5. Removal of retained products of conception (RRP)	
#6. Assisted vaginal delivery (AVD)	
#7. Neonatal resuscitation with bag and mask	

EmONC: emergency obstetric and newborn care

Source: Monitoring emergency obstetric care: a Handbook. WHO; 2009

The Inter-Agency Working Group for Reproductive Health in Crises (IAWG) guidelines place a priority on ensuring optimal provision of EmONC services during post-conflict recovery to address excess mortality in mothers and newborns [24]. A clear understanding of the capacity of the existing health system to respond to the reproductive health needs of affected populations is essential for effective program planning and implementation, especially with regard to safe motherhood [25]. For example, the IAWG advocates the use of a well-established Needs Assessment Toolkit [26] that allows for a system of EmONC process/performance evaluation against United Nations (UN) standards, which is instrumental in identifying gaps and guiding programmes.

From 2006 to 2013, state-based conflict (i.e., armed fighting between a state and an opposition group resulting in at least 25 battle-related deaths in a year) disproportionately affected the eastern Democratic Republic of the Congo (DRC) in general and the North Kivu Province (NKP) in particular [27]. During that period, the North Kivu Province witnessed 98 of the 128 conflict events that occurred across the country, opposing government troops against foreign-backed rebel groups [27]. As a reflection of the impact of conflict on public health, the east of DRC recorded higher preventable mortality, including excess neonatal mortality, than in the west [28, 29]. In the North Kivu Province, maternal mortality was as high as 790 deaths per 100,000 live births for the first half of 2013 [30]. Recent evidence indicates that proximity to and deadliness and duration of armed conflict in SSA are associated with indirect effects on the survival of mothers and their babies; these effects persist for years after the conflict has ceased [15, 31]. This underscores the need for sustained and informed measures in revitalizing essential health services, including EmONC.

However, in the North Kivu Province, there is a lack of data on the post-conflict status of EmONC. This situation can be explained in part by poor government stewardship and ‘the proliferation of fragmented humanitarian and recovery initiatives’, factors previously observed in other post-conflict contexts in SSA (South Sudan and Sierra Leone) and eastern DRC [32–35]. These often lead to uncoordinated health interventions, with little attention to data-driven plans and policies that are customized to local contexts [36, 37]. Therefore, this study aimed to contribute to filling this gap by assessing the availability, use and quality of EmONC in 3 Health Zones (HZs) in the North Kivu Province of eastern DRC. Evidence generated from this study will contribute to informed programming and data-driven interventions, involving local and external stakeholders, as part of the post-conflict effort to improve maternal and neonatal health in the Province.

## Methods

### Study design and setting

This is a cross-sectional survey at health facility level conducted from March to May 2017 in the North Kivu Province (NKP). From 2006 to 2013, this Province accounted for about two thirds of battle-related deaths across the country, reflecting the intensity of conflict events between government troops and two major rebel groups backed by neighbouring countries (the *Congrès National pour la Défense du Peuple* [CNDP] and *the Mouvement du 23 Mars* [M23]) [27]. The NKP counts 34 health zones (HZ) out of the 515 nationwide, serving urban, rural or urbano-rural administrative areas with at least 100,000 inhabitants and a maximum diameter of 150 km [38].

The HZ is a decentralized entity that constitutes the operational unit for planning and implementing health services in DRC. It operates as a tiered system consisting of a network of health facilities, supervised by the Central Bureau of the Health Zone (CBHZ), with increasing service capacity along a chain of referral [38]. Health centres (HC) are first-line facilities dedicated to primary health care tasks (including the provision of antenatal care and the management of normal deliveries), delegated to a team of versatile nurses. They refer cases that are beyond their means and competencies to a referral health centre (RHC) or the referral hospital (RH).

Three health zones (HZs), namely Goma, Karisimbi, and Rutshuru, were purposively selected based on their ‘red flag’ proximity—that is, within a 50 km range [15, 31], to most of the deadliest conflict events from 2006 to 2013 [27, 39], and based on their position across the urban/rural spectrum in the North-Kivu Province to represent the status of maternal and neonatal health care in urban (Goma), urbano-rural (Karisimbi) and rural (Rutshuru) post-conflict settings.

The HZs of Goma and Karisimbi integrate both state-owned and private not-for-profit (religious or other) referral facilities (RHs and RHCs) into the public health sector. Not-for-profit organizations (NFPO) have entered into a contractual agreement with the state specifying the rights and duties of both partners; however, they remain largely autonomous in terms of management.

### Selection of health facilities and sample size

We selected only public health facilities (state-owned and private not-for-profit) providing maternal and neonatal health (MNH) care across the 3 HZs. The rationale for this was that institutional births represent 92% of deliveries in North Kivu province, of which 80% take place in public facilities [40]. A complete list of facilities with maternal services, including HCs, RHCs, and RHs, was provided by the respective CBHZs. Therefore, all 42 public facilities providing MNH services in the 3 HZs were included in the study. Table 2 shows these health facilities per HZ and per level. As per the DRC Ministry of Health (MOH) guidelines, all HCs and referral facilities (RHs and RHCs) are required to provide basic and comprehensive EmONC 24 h a day and 7 days a week, respectively.

**Table 2:** Public health facilities providing maternal and neonatal care per level and per study Health Zones (HZ) in the North-Kivu province of Eastern Democratic Republic of the Congo, 2017

Health Zone (HZ)	Population	Expected number of live births*	Referral hospitals (RH)	Referral health centres (RHC)	Health centres (HC)
Goma	243,685	9869	Hôpital Provincial du Nord-Kivu^†^ Hôpital Général Charité Maternelle^†^	CSR Carmel^†^ CSR Heal Africa^†^ CSR Keshero^†^ CH Bethesda^†^	CS Mapendo CS Buhimba CS Casop CS Afia Himbi CS Kasika
Karisimbi	461,089	21,164	Hôpital Général Virunga Hôpital Militaire Régional	CSR Kahembe CSR Albert Barthel CH Notre dame d’Afrique CH La Résurrection	CS Murara CS Bujovu CS Majengo CS Lubango CS La solidarité CS Virunga CS Mabanga CS Katoyi
Rutshuru	283,432	13,009	Hôpital Général de Rutshuru^‡^	CSR Kinyandonyi CSR Kiwanja CSR Mapendo CSR Rubare CSR Vitshumbi	CS Rutshuru CS Biruma CS Buturande CS Kalengera CS Kakomero CS Katale CS Kibututu CS Mabungo CS Murambi CS Rugari CS Umoja
Total	988,206	44,042	5	13	24

Source: Central bureau of the Health Zones (CBHZ)

*calculated as population x crude birth rates (CBR, as reported in the 2013–2014 DRC Demographic Health Survey [42])

CSR: centre de santé de référence, CH: centre hospitalier, CS: centre de santé

^†^Non-for-profit organisations including 5 faith-based and 1 NGO owned (CSR Heal Africa) health facilities

^‡^ Public RH supported by Médecin Sans Frontières

### Data collection

Data were obtained from medical records and registries for the year 2016, as well as from interviews with relevant in-charges and observation of available equipment and medical supplies, using the EmONC Needs Assessment (NA) toolkit. This toolkit is organised in modules of questionnaires developed and refined over time by the Averting Maternal Death and Disability (AMDD) programme at Columbia University Mailman School of Public Health. The WHO, UNFPA, and UNICEF have adopted these modules to capture key indicators of availability, use, and quality of EmONC services. These indicators are constructed to inform interventions aiming to reduce maternal and neonatal mortality by identifying gaps, monitoring implementation, and measuring progress [26]. Moreover, these modules have been proven useful in conflict-affected settings [41].

Prior to the survey, we held meetings with the Chief Medical Officer (CMO) and the CBHZ team of each HZ. During these meetings, the French versions of the AMDD NA modules were discussed and adapted to suit the local context, guided by the research objectives. Adaptations included editing/deleting some questions and response options. The CMOs granted authorizations to collect data at the surveyed facilities and provided relevant information on public health facilities (population covered, ownership/management).

Four research assistants, all final year students at the Midwifery Section of the Institut Supérieur des Technique Médicales de Goma, were recruited. They received 3-day training to ensure a clear understanding of the objectives/methods of the survey, the content of the modules and how to fill them out. Didactic sessions covered proper interviewer behaviour, relevant ethical considerations, and appropriate identification of direct obstetric complications (ante- and post-partum haemorrhage, severe pre-eclampsia/eclampsia, obstructed labour, ectopic pregnancy, and postpartum sepsis) using the WHO definitions [26].

In each HZ, the Principal Investigator (PI) organized a data collection schedule in consultation with the heads of health facilities. The PI supervised data collection, which followed a top-down order in every HZ (i.e., RHs first, then RHCs and HCs). Data on deliveries, pregnancy-related and childbirth complications, maternal, foetal and early neonatal outcomes, and procedures related to EmONC were extracted from facility registries, procedure reports, and from patient records when necessary. Interviews were conducted with heads of facilities (medical doctors in referral facilities and registered nurses in HCs) and in-charges of units of interest (maternity ward, delivery room, operation theatre, pharmacy, and laboratory), or with a direct collaborator. They focused on the provision of signal functions during the last 3 months, the number and composition of health staff, and the availability of equipment and medical supplies. Facility walk-throughs were carried out to observe the availability of medical supplies, facilities, and equipment.

### Data management and analysis

During data collection, a number was attributed to each facility and written at the top of every page of each module, starting with RH, then RHC and HC, in Goma, Karisimbi, and Rutshuru, respectively. EpiData 3.1 was used to create data entry fields with in-build checks and each module had its own file. Data were double entered by 2 trained data entry operators and discordances were sorted out by revisiting the respective questionnaires.

Data keyed in using EpiData were exported to Stata 15 for data handling and analysis. Descriptive statistics including means and medians for continuous variables, and frequencies and proportions for categorical variables were performed as appropriate. Analyses were guided by the Handbook on Monitoring Emergency Obstetric Care by UN partners and the AMDD programme to assess the performance of signal functions and calculate indicators of availability, use, and quality of EmONC services in these health zones. Estimates of the expected live births were used to compute EmONC indicators.

### Ethical considerations

This study was approved by Kyoto University Graduate School and Faculty of Medicine, Ethics Committee, as well as by the Ethics Committee of the University of Kinshasa School of Public Health.

## Results

### Profile of surveyed health facilities and volume of deliveries

Of the 42 public health facilities surveyed in the 3 HZs, 24 were health centres, all owned and managed by the state, and 18 were referral institutions, of which 5 were RHs and 13 were RHCs (Table 2). In the HZ of Goma, most referral facilities (5/6; 83%) were owned and managed by private non-for-profit organizations (NFPO) including 1 RH by the Catholic church, 3 RHCs by Protestant churches, and 1 RHC by a non-governmental organisation. In the HZ of Karisimbi, half of the referral facilities (3 RHCs) were faith-based organisations managed by Protestant churches. All referral facilities in the HZ of Rutshuru were state owned. However, as part of the humanitarian response to conflict-related health crises, the RH in the HZ of Rutshuru was assisted and co-managed by Médecins Sans Frontières (MSF), dating back to 2005. MSF built a maternity-waiting home near the RH and, among other things, offered full exemption from user fees for all CS and charged a flat-fee of USD 5 for vaginal deliveries.

In 2016, the surveyed facilities conducted 35,283 deliveries: 7824 in Goma, 14.997 in Karisimbi, and 12,467 in Rutshuru. Of note, not-for-profit referral facilities (RHs and RHCs) attended 45.8% (10,459/22,821) of deliveries and 73.2% (2446/3337) of CS in the HZs of Goma and Karisimbi, while 36.7% (4578/12,462) of deliveries and 83% of CS (1491/1797) in the HZ of Rutshuru took place at the RH.

### Provision of signal functions

Only 17% (3/18) of the referral facilities (the faith-based RH and 1 faith-based RHC in Goma, and the RH in Rutshuru) provided the nine signal functions within the last 3 months prior to the survey and thus met the criteria for functioning cEmONC facilities (see Table 3). All the remaining (83%; 15/18) were partial cEmONC facilities (i.e., 8 or fewer signal functions provided). Of these, 20% (3/15) were short of a signal function (AVD) to qualified as functioning cEmONC facilities, which included 1 faith-based RHC and the government RH in Goma, and 1 faith-based RHC in Karisimbi.

**Table 3:** Signal functions performed by designated EmONC facilities per level in the 3 health zones (HZs) in the North-Kivu Province of Eastern Democratic Republic of the Congo (DRC), 2017

Health zones	#1. Antibiotics	#2. Oxytocics	#3. Anti-convulsants	#4. MRP	#5. RRP	#6. AVD	#7. Neonatal resuscitation	#8. Caesarean section	#9. Blood transfusion	Facilities performing all signal function*	Facilities providing services 24 h a day and 7 days a week
	n(%)	n(%)	n(%)	n(%)	n(%)	n(%)	n(%)	n(%)	n(%)	n(%)	n(%)
**Goma**											
RH and RHC (*n* = 6)	6 (100)	6 (100)	6 (100)	6 (100)	6 (100)	2 (33.3)	6 (100)	6 (100)	5 (83.3)	2 (33.3)	6 (100)
HC (*n* = 5)	5 (100)	5 (100)	3 (60.0)	5 (100)	4 (80.0)	0 (0)	3 (60.0)	NA	NA	0 (0)	0 (0)
**Karisimbi**											
RH and RHC (n = 6)	6 (100)	6 (100)	3 (50.0)	6 (100)	6 (100)	0 (0)	6 (100)	6 (100)	4 (66.7)	0 (0)	6 (100)
HC (*n* = 8)	5 (62.5)	8 (100)	0 (0)	8 (100)	2 (25.0)	0 (0)	1 (12.5)	NA	NA	0 (0)	0 (0)
**Rutshuru**											
RH and RHC (n = 6)	4 (66.7)	6 (100)	2 (33.3)	5 (83.3)	5 (83.3)	1 (16.7)	2 (33.3)	4 (66.7)	1 (16.7)	1 (16.7)	4 (66.7)
HC (*n* = 11)	7 (63.6)	11 (100)	0 (0)	11 (100)	1 (9.1)	0 (0)	1 (9.1)	NA	NA	0 (0)	0 (0)
**All HZs**											
RH and RHC (*n* = 18)	16 (88.9)	18 (100)	11 (61.1)	17 (94.4)	17 (94.4)	3 (16.7)	14 (77.8)	16 (88.9)	10 (56)	3 (16.7)	16 (88.9)
HC (*n* = 24)	17 (70.8)	24 (100)	3 (12.5)	24 (100)	7 (29.2)	0 (0)	5 (20.8)	NA	NA	0 (0)	0 (0)
Total (*n* = 42)	33 (78.6)	42 (100)	14 (33.3)	41 (97.6)	24 (57.1)	3 (7.1)	19 (45.2)	16 (88.9†)	10 (55.6†)	3 (7.1)	16 (38.1)

MRP: manual removal of placenta, RRP: removal of retained placenta, AVD: assisted vaginal delivery

HC: health centres, RHC: referral health centre, RH: referral hospital

* #1-#7 for HC and #1-#9 for R and RHC

†Calculated with the total number (n = 18) of RH and RHC as the denominator

NA: not applicable

Among the surveyed health centres (HCs), none performed all the 7 bEmONC signal functions during the last three months, nor could they offer EmONC services 24 h, 7 days a week (24/7). Most HCs in Rutshuru (91%, 10/11) and Karisimbi (88%;7/8) performed only 1 to 3 signal functions of bEmONC, whereas in Goma, 80% (4/5) of HCs performed 4 to 6 basic signal functions, of which two could have qualified as functioning bEmONC facilities had they performed AVD.

Assisted vaginal delivery (AVD) was the least performed signal function (7.1%;3/42), followed by parental administration of anticonvulsants (33.3%;14/42) and neonatal resuscitation (45.2%; 19/42) (Table 3). ‘Policy issue’ (i.e., HZ or facility policies not allowing the performance of a signal function) was the reason reported by about three-quarters (74.3%; 29/39) of facilities that did not perform AVD, including all HCs in Rutshuru, 87.5% (7/8) of HCs in Karisimbi, and 60% (3/5) of HCs in Goma. Similarly, ‘no indication’ (i.e., no patient needing a signal function came to the facility) was the reason given by 71.4% (20/28) and 69.5% (16/23) of facilities that did not perform parenteral administration of anticonvulsants and neonatal resuscitation, respectively. Most of the facilities that did not indicate parenteral anticonvulsants (75%;15/20) and neonatal resuscitation (81.3%;13/16) were HCs.

Further analyses showed that nearly all the facilities that reported ‘no indication’ for parenteral administration of anticonvulsants (95.0%; 19/20) didn’t provide this signal function even during the past 12 months. Also, 62.5% (10/16) and 50.0% (4/8) of facilities that didn’t provide neonatal resuscitation (NR) and AVD due to no indication, respectively, didn’t have the necessary equipment to perform these signal functions (i.e., neonatal bag and mask for NR and vacuum extractor or forceps for AVD).

### Indicators of EmONC

The existing public health facilities designated to provide EmONC services exceeded the minimum number of EmONC delivery points needed per 500,000 population in the 3 HZs (Table 4). However, none of the health zones achieved the minimum number of functioning EmONC facilities as per the WHO recommendations (at least 5 EmONC facilities for every 500,000 population with at least 1 cEmONC facility), with an unmet need less pronounced in the HZ of Goma (Tables 4 and 5). In other words, the number of functioning EmONC facilities per 500,000 population in the study HZs was 1.5.

**Table 4:** Distribution of designated EmONC facilities in the study health zones (HZs) in the North-Kivu Province of eastern Democratic Republic of the Congo (DRC) in comparison with the minimum number of functioning EmONC facilities required by the World Health Organization (WHO), 2017

Health zones	Total population	Minimum number of EmONC facilities for 500,000 population as per the WHO^†^[a]	Number of designated cEmONC facilities per 500,000 population in the study HZs^‡^	Number of designated bEmONC facilities per 500,000 population in the study HZs^‡^	cEmONC facilities		bEmONC facilities		Functioning EmONC facilities as a proportion of the minimum number recommended by the WHO ([b + c]/a, %)
					Minimum number acceptable by the WHO	Number of functioning cEmONC facilities in the study HZs [b]	Minimum number acceptable by the WHO	Number of functioning bEmONC facilities in the study HZs [c]	
Goma	243,685	3	12	10	1	2	2	0	66.7
Karisimbi	461,089	5	7	9	1	0	4	0	0.0
Rutshuru	283,432	3	11	19	1	1	2	0	33.3
All HZs	988,206	10	9	12	2	3	8	0	30.0

cEmONC: comprehensive emergency obstetric and newborn care, bEmONC: basic emergency obstetric and newborn care

†Calculated as population/500,000 × 5 (rounded up)

‡Calculated as 500,000/population x number of designated EmONC or bEmONC facilities

**Table 5:** Indicators of EmONC in the study health zones (HZs) in the North-Kivu Province of Eastern Democratic Republic of Congo (DRC), 2016–2017

Indicators	Description*	HZ of Goma			HZ of Karisimbi			HZ of Rutshuru			Overall		
		MAL	LFF	DAF (n = 11)	MAL	LFF	DAF (*n* = 14)	MAL	LFF	DAF (*n* = 17)	MAL	LFF	DAF (n = 42)
Availability of functioning EmONC	At least 5 functioning EmONC facilities per 500,000 population including with at least 1 functioning cEmONC facility.	3	2	NA	5	0	NA	3	1	NA	10	3	NA
Proportion of all births in functioning EmONC facilities	Proportion of births in functioning EmONC facilities among expected number of births in a population^a^	15%	17.8% (1753/9869)	79.3% (7824/9869)	15%	_	70.9% (14,997/21,164)	15%	35.2% (4578/13,009)	95.8% (12,462/13,009)	15%	14.4% (6331/44,042)	80.1% (35,283/44,042)
Met need for EmONC	Proportion of women with direct obstetric complications (DOC) treated in functioning EmONC facilities among all expected number of women with DOC ^b^	100%	6.6% (97/1480)	17.2% (254/1480)	100%	_	4.9% (154/3175)	100%	5.4% (105/1951)	7.0% (137/1951)	100%	3.1% (202/6606)	8.3% (545/6606)
Caesarean sections as a proportion of all births	Proportion of caesarean section occurring in EmONC facilities among expected number of live births in a population^c^	5–15%	7.2% (715/9869)	22.9% (2261/9869)	5–15%	_	5.1% (1076/21,164)	5–15%	11.5% (1491/13,009)	13.8% (1797/13,009)	5–15%	5.0% (2206/44,042)	11.7% (5134/44,042)
Direct obstetric case fatality rate (DOCFR)	Proportion of women with MDOC who died in EmONC facilities	< 1%	5.2% (5/97)	4.7% (12/254)	< 1%	_	7.4% (9/121)	< 1%	3.8% (4/105)	5.1% (7/137)	< 1%	4.5% (9/202)	5.1% (28/545)
Intrapartum and very early neonatal death rate (INDR)	Proportion of births that result in a very early neonatal death or an intrapartum death among the births occurring in functioning EmONC facilities	‡	1.2% (21/1753)	2.1% (167/7824)	‡	_	1.7% (258/14,997)	‡	2.0% (95/4578)	0.9% (107/12,462)	‡	1.9% (119/6331)	1.5% (532/35,283)

*Source: Monitoring emergency obstetric care: a handbook. WHO, 2009 [26]

EmONC: emergency obstetric and newborn care, cEmNOC: comprehensive EmONC, MAL: minimum acceptable level, LFF: Level met by functioning facilities, DAF: data for all facilities

^a^ Calculated as number of births in functioning EmONC/expected live births × 100 (for expected live birth see Table 2)

^b^ Expected number of women with MDOC was calculated multiplying 15% with expected number of births in Table 2

^c^ Calculated as number of CS/expected number of births × 100

‡ Standard to be determined

NA: not applicable, _: not calculated

Overall, 14.3% (6331/44,042) of expected births in 2016 occurred in functioning EmONC facilities (Table 5). In the HZ of Rutshuru, this proportion (35.2%; 4578/13,009) was about twice as high as in the HZ of Goma (17.8%; 1753/9869). Deliveries in surveyed facilities in the 3 HZs (i.e., institutional deliveries) represented 80.1% (35,283/44,042) of births in 2016. In Goma, Karisimbi, and Rutshuru, the institutional delivery rate was 79.3% (7824/9869), 70.9% (14,997/21,164), and 95.8% (12,462/13,009), respectively (Table 5).

The proportion of MDOC managed in functioning EmONC facilities was only 3.1% (202/6606) overall (Table 5). This proportion was 6.6% (97/1480) in the HZ of Goma and 5.4% (105/195) in the HZ Rutshuru. In contrast, the proportion of births by caesarean sections that took place in functioning EmONC facilities was 5.0% (2206/44,042) overall; 7.2% (715/9869) in the HZ of Goma and 11.5% (1491/13,009) in the HZ of Rutshuru. Caesarean deliveries in all referral facilities surveyed (i.e., population-based caesarean section rate [PCSR]) represented 11.7% (5134/44,042) of expected births. In Goma, Karisimbi, and Rutshuru, the PCSR was 22.9% (2261/9869), 5.1% (1076/21,164), and 13.8% (1797/13,009), respectively (Table 5).

With regards to the quality of EmONC, the direct obstetric case fatality rate (DOCFR) and the intrapartum and very early neonatal death rate (INDR) in EmONC facilities were 4.5% (9/202) and 1.9% (119/6331), respectively (Table 5). The DOCFR in Goma was higher (5.2%;5/97) than that in Rutshuru (3.8%;4/105) and the INDR (1.2%;21/1753) about half of that in Rutshuru (2.0%;95/4578). When including all facilities surveyed, the INDR was 1.5% (532/35,283), with very little variation between the HZs, and the DOCFR was 5.1% (28/545). The highest DOCFR was in Karismbi (7.4%; 9/121). Of note, all direct obstetric deaths occurred in referral facilities. Major direct obstetric complications (MDOC) by causes and related maternal deaths in the study HZs are shown in Table 6.

**Table 6:** Causes of Major Direct Obstetric Complications (MDOC) and related deaths in the 3 health zones (HZ) in the North-Kivu Province of Eastern Democratic Republic of Congo (DRC), 2016

MDOC diagnosed in the facilities surveyed	Occurrences of MDOC by causes n (%)	Deaths related to MDOC n (%)
Ante or postpartum haemorrhage	261 (47.9)	14 (50.0)
Retained placenta	6 (1.1)	1 (3.6)
Ruptured uterus	56 (10.3)	4 (14.3)
Postpartum sepsis	82 (15.0)	4 (14.3)
Severe pre−/eclampsia	95 (17.4)	3 (10.7)
Abortion complications	40 (7.3)	1 (3.6)
Ectopic pregnancy	5 (0.9)	1 (3.6)
Total	545 (100)	28 (100)

### Staff, supplies and equipment for EmONC

Table 7 shows the staff available for EmONC in the surveyed facilities per HZ and per level of facilities. Overall, nurses represented the largest category of staff (62.6%;325/519), followed by physicians (25.0%; 130/519) and midwives (12.3;64/519). In line with the MOH guidelines, HCs were staffed only with non-physician practitioners (nurses or midwives). All HCs had at least 2 nurses, the minimum number set in national guidelines, with only a third of them (33.3%, 8/24) having midwives on staff.

**Table 7:** Availability of staff for emergency obstetric and newborn care (EmONC) services per level of facilities and per study health zones (HZ) in the North-Kivu Province of Eastern Democratic Republic of Congo (DRC), 2017

Health zones and facilities	Staff for EmONC services				
	All	Nurses	Midwives*	Medical doctors	
**Overall**	519	325	64 ^a^	130	
				Specialists†	General practitioners
RF (n = 18)	384	204 (62.8^‡^)	50	17	113
HC (n = 24)	135	121 (37.2^‡^)	14	NA	NA
Median number per facility	_	6 (IQR 4–8)	2 (IQR 1.5–4)	3.5^‡^ (IQR 2–10)	
**Goma**	198	96	25 ^b^	77	
RF (n = 6)	163	63 (65.6^‡^)	23	16	61
HC (n = 5)	35	33 (34.4^‡^)	2	NA	NA
Median number per facility	_	7 (IQR 6–12)	4 (IQR 2–4)	9.5^‡^ (IQR 8–13)	
**Karisimbi**	150	102	19 ^c^	29	
RF (n = 6)	105	63 (61.8^‡^)	13	0	29
HC (n = 8)	45	39 (38.2^‡^)	6	NA	NA
Median number per facility	_	7 (IQR 5–9)	2 (IQR 1–3)	3^‡^ (IQR 2–8)	
**Rutshuru**	171	127	20 ^d^	24	
RF (n = 6)	116	78 (61.4^‡^)	14	1	23
HC (n = 11)	55	49 (38.6^‡^)	6	NA	NA
Median number per facility	_	4 (IQR 3–5)	2 (IQR 1.5–3.5)	2^‡^ (IQR 2–3)	

HC: health centres, RF: referral facilities (referral hospitals and referral health centres), IQR: interquartile range

^‡^Percentages of nurses in the study health zones per level of facilities

^‡^Obstetricians and paediatricians

*Not available in 18 facilities (4 HCs in Goma, 5HCs in Karisimbi, 2 RHCs and 7 HCs in Rutshuru)

^a^ RF (*n* = 16), HC (n = 8), ^b^ RF (n = 6), HC (n = 1)7, ^c^ RF (*n* = 6), HC (*n* = 3), ^d^ RF (n = 4) HC (n = 4)

^‡^In RH and RHC only

NA: not applicable, _: not calculated

Referral facilities employed a mix of cadres, including physicians (general practitioners and/or specialists), nurses, and midwives. Nearly all (94.1%;16/17) of the specialists (obstetricians and paediatricians) and over half (54.0%;61/113) of the general practitioners worked in Goma. Most of the midwives (82.8%;24/29) and specialists (75.0%;12/16) in the HZs of Goma and Karisimbi were NFPO health workers. In the HZ of Rutshuru, 58.3% (14/24) of physicians were working at the referral hospital.

On average, there were fewer nurses on staff in the HCs located in the HZ of Rutshuru (median = 4, IQR  4–5) than in those located the HZs of Goma (median = 7, IQR  6–7) and Karisimbi (median = 7.5, IQR  6.5–9). The median number of medical doctors in referral facilities was higher in the HZ of Goma (9.5, IQR 8–13) than in the HZs of Karisimbi (3, IQR 2–8) and Rutshuru (2, IQR 2–3).

Regarding the availability of essential drugs, equipment in the facilities surveyed, magnesium sulphate was the least available drug in the facilities surveyed (45.2%;19/42). Vacuum extractors were the least available equipment (21.4%;9/42), followed by filled oxygen cylinders and neonatal intravenous fluid sets (Table 8). These drugs and equipment were more frequently unavailable in HCs located in the HZs of Karisimbi and Rutshuru. There was a pattern whereby essential drugs and equipment for EmONC were more likely to be available in facilities located in the HZ of Goma. In the HZ of Rutshuru, 33% (2/6) of the referral facilities, and 36% (4/11) of the HCs did not have an operational laboratory because of a lack of equipment or technicians.

**Table 8:** Availability of essential drugs, equipment, ambulance and communication means in the facilities surveyed in the 3 health zones (HZs) in the North-Kivu Province of Eastern DRC, 2017

Drugs, equipment and other infrastructures	All facilities (*n* = 42)	HZ of Goma		HZ of Karisimbi		HZ of Rutshuru	
		RH and RHC (n = 6)	HC (n = 5)	RH and RHC (n = 6)	HC (n = 8)	RH and HC (n = 6)	HC (n = 11)
	n(%)	n(%)	n(%)	n(%)	n(%)	n(%)	n(%)
**Drugs**							
Injectable beta-lactams	42 (100)	6 (100)	5 (100)	6 (100)	8 (100)	6 (100)	11 (100)
Injectable metronidazole	33 (78.6)	6 (100)	5 (100)	6 (100)	6 (75.0)	6 (100)	4 (36.4)
Magnesium sulphate	19 (45.2)	6 (100)	3 [43]	6 (100)	2 (25.0)	5 (83.3)	0 (0)
Diazepam	39 (92.8)	6 (100)	3 (60.0)	6 (100)	8 (100)	6 (100)	10 (90.9)
Anti-hypertensive drugs	26 (61.9)	6 (100)	2 (40.0)	6 (100)	6 (75.0)	5 (83.3)	1 (9.1)
Oxytocics	42 (100)	6 (100)	5 (100)	6 (100)	8 (100)	6 (100)	11 (100)
Crystalloid fluids	42 (100)	6 (100)	5 (100)	6 (100)	8 (100)	6 (100)	11 (100)
**Equipment**							
Foetal stethoscope	42 (100)	6 (100)	5 (100)	6 (100)	8 (100)	6 (100)	11 (100)
Blood pressure cuff	42 (100)	6 (100)	5 (100)	6 (100)	8 (100)	6 (100)	11 (100)
Filled oxygen cylinder	12 (28.9)	6 (100)	0 (0)	3 (50.0)	0 (0)	3 (50.0)	0 (0)
Catheter for IV line	40 (95.2)	6 (100)	5 (100)	6 (100)	7 (87.5)	6 (100)	10 (90.9)
Uristix	26 (61.9)	3 (50.0)	2 (40.0)	6 (100)	5 (62.5)	6 (100)	4 (36.4)
Neonatal IV fluid set	22 (52.4)	6 (100)	3 (60.0)	6 (100)	3 (37.5)	4 (66.7)	0 (0)
Neonatal bag and mask	27 (64.3)	6 (100)	2 (40.0)	6 (100)	4 (50.0)	6 (100)	3 (27.3)
Kit for uterine evacuation	28 (66.7)	4 (66.7)	4 (80.0)	5 (83.3)	4 (50.0)	6 (100)	5 (45.5)
Aspirator	35 (83.3)	6 (100)	5 (100)	6 (100)	7 (87.5)	6 (100)	5 (45.5)
Vacuum extractor	9 (21.4)	6 (100)	1 (20.0)	1 (16.7)	0 (0)	1 (16.7)	0 (0)
Resuscitation table	35 (83.3)	6 (100)	4 (80.0)	6 (100)	8 (100)	6 (100)	5 (45.5)
**Other infrastructures**							
Ambulance†	10 (23.8)	3 (50.0)	0 (0)	3 (50.0)	0 (0)	4 (66.7)	0 (0)
Communication means for referral‡	16 (38.1)	6 (100)	0 (0)	6 (1009	0 (0)	3 [44]	1 [9]

RH: referral hospital, RHC: referral health centre, HC: health centre, IV: intravenous

†Car or motorcycle

‡Cell phone or two-way radio

### Transport and communication means for referral

The ten facilities with a functional ambulance (car or motorcycle) on-site were all referral facilities, including two thirds of referral facilities in Rutshuru (4/6) and half of the referral facilities both in Goma and Karismbi (**Tale 7**). They were all charging fees for the use of the ambulance when an institutional referral was requested, except for the referral hospital of Rutshuru where MSF took care of the ambulance operations. Relatedly, all the referral facilities in Goma and Karisimbi and 50% (3/6) of those in Rutshuru had a means of communication (cell phone or two-way radio) for referral purposes, in stark contrast with almost none of the HCs (4.1%, 1/24).

## Discussion

According to the World Health Organization, the delivery of all EmONC services over the past 3 months by at least 5 facilities serving 500,000 inhabitants indicates that pregnancy-related complications are managed by trained personnel, medical equipment and supplies are sufficient, and skills are maintained [26]. Our findings show that only 30% of the minimum acceptable number EmONC facilities was met, with an availability gap less marked in the HZ of Goma than in the HZs of Rutshuru and Karisimbi, respectively. Relatedly, the process indicators related to the use and quality EmONC indicate that study HZs fell short of the WHO standards, except for the proportion of births by caesarean section. Resources needed for EmONC and institutional referral were scarce in surveyed HCs.

None of the surveyed HCs could offer EmONC 24 h a day and 7 days a week (24/7). It is conceivable that this finding is related to the impact of residual insecurity that characterize post-conflict settings [42]. This situation impedes patients’ admission or referral within the health system and tend to limit the availability of skilled health personnel to provide EmONC 24/7, especially in primary healthcare facilities, as suggested in previous studies in post-conflict SSA [43–45]. A high priority needs to be placed on further addressing residual insecurity in the 3 HZs, which is paramount in reducing preventable mortality in conflict-affected populations [29].

Very few of the facilities surveyed performed all the 7 basic signal functions in the past 3 months, with AVD being the least frequently performed. This parallels the findings of a previous survey on the provision of key health services in a national sample of health facilities in DRC. It revealed that less than 12% of those offering maternity services were performing all basic EmONC services [46]. Also, the finding that AVD was the least likely signal function to be reported, especially in HCs, is consistent with existing literature on the subject in SSA [47, 48].

If we were to ignore AVD, the 3 HZs could meet the target of at least 5 functioning EmONC facilities for 500,000 people. While most facilities couldn’t offer this signal function on policy grounds (i.e., HZ or facility policies not allowing the performance of a signal function), half of the facilities that reported ‘no indication’ didn’t have a vacuum extractor for instrumental vaginal delivery. Restrictive policies targeted mainly HCs regardless of their location, illustrating a perceived lack of trained operators at that level of care. Moreover, 50% of facilities that owned a vacuum extractor did not indicate any AVD, which might reflect unfamiliarity with this technique. Lack of equipment and limited staff training and exposure are common obstacles to the performance of AVD in resource-poor settings [49–52]. Similarly, restrictive policies and the lack of equipment/drugs were the main reasons why neonatal resuscitation and parenteral administration of anticonvulsants could not be performed.

Restrictive policies raise concerns as to training and practice opportunities for health providers, especially those working in HCs. These restrictions, along with a lack of equipment, compromise chances for health providers to acquire skills and contribute to maintaining a situation where targeted services remain virtually unavailable at the primary health care level [50]. In line with WHO guidelines [53], interventions prioritising competency-based training for frontline providers and the use of simple and cost-effective equipment have been proved effective [54, 55]. Such programming could offer a practical alternative to restrictive policies and promote better access to quality primary health care, which is a key step toward post-conflict health recovery [43, 56].

In Karisimbi and Rutshuru, none of the HCs administered anticonvulsants and, very few had stocked magnesium sulphate, the anticonvulsant of choice, shown to avert up to 85% of severe pre−/eclampsia (SPE) related deaths and disabilities [57]. This suggests that it was very likely for a pregnant woman presenting with SPE to be referred without being administered the loading dose of magnesium sulphate (10 mg). In rural Bangladesh, Shamsuddin et al. found better maternal and perinatal prognoses in pregnant women who received the loading dose before referral than in those that did not [58].

Health facilities in Goma outperformed those in Karisimbi and Rutshuru in terms of service provision, overall. The performance of more EmONC signal functions in urban settings has already been reported in SSA [59] and in South-East Asia [60], a disparity that might illustrate an imbalance in the procurement of medical supplies and equipment, in training opportunities, and in the posting and retention of skilled health staff. Remarkably, two of the three functioning cEmONC facilities catered to the urban population, which might imply better access to comprehensive life-saving care for pregnant women in Goma. Of note, the presence of a functioning cEmONC facility in Rutshuru is consistent with a previous study in rural humanitarian settings in the North Kivu province of eastern DRC [61].

The inadequacies of the EmONC services found in state-run facilities in the study HZs are concordant with previous literature in DRC [61–63]. They appear to be systemic in nature and might reflect the level of public resource allocation for the health system. Available evidence points to this analysis. The DRC government expenditure on health (USD 12–13 per capita) is one the lowest in SSA [64], with a health budgetary allocation (4–5%) far below the 2001 Abuja declaration target of at least 15% of the national budget to be allocated to the health sector to ensure universal care coverage [65, 66]. Also, studies in DRC showed other factors related to persistent fragility that create bottlenecks in the functioning of the health system, including dependence on user fees and fragmented/vertical multi-donor inputs, inefficient budget preparation approaches, unmonitored budget execution, and poor governance [67–69]. Previously identified barriers to the delivery of EmONC in post-conflict SSA included systemic and human resource factors such as limited infrastructures and procurements, insignificant and erratic pay and poor living and working conditions of health workers [44].

Our results show that about half of facility births in the HZs of Goma and Karisimbi took place in referral NFPOs and that these facilities hired a higher proportion of health staff in both HZs and performed more signal functions. This tends to reflect how the availability of enough resources translates into adequate functionality of health facilities and points to women’s self-referral behaviour to seek maternal care where better quality is perceived, as shown in previous work [70–72]. A study by Tabatabai P et al. in southern Tanzania comparing faith-based organisations (FBO) and public hospitals found that maternal health service capacity was more appropriate in FBOs [73]. In addition, the number of EmONC functions performed during a given time period has been shown to have an effect (i.e, for each additional EmONC SF available, bypassing odds decrease substanatially) on bypassing frontline facilities for childbirth care [71, 74]. However, bypassing frontline facilities has an incidence on the number of cases of deliveries and pregnancy-related complications primary care health workers can see and manage in a given time period, perpetuating low EmONC functionality [75].

In the rural HZ of Rutshuru, the RH attended over a third of deliveries and four fifths of CS and hired the majority of health workers across the HZ. These findings reflect the impact of humanitarian assistance through financial and technical support that this RH was receiving from MSF. For example, in a rural conflict-affected district in Afghanistan, Lagrou D et al. found a steady increase in caseload at a cEmONC facility run by MSF [76]. In Rutshuru, MSF offered user fees exemption for CS and a maternity-waiting home, measures proven to address inequities in childbirth care [77, 78]. However, this assistance comes with potential downsides. First, by absorbing the majority of cases within a health district, humanitarian-assisted hospitals deprive other facilities, particularly primary care ones, of being exposed to case management, undermining the acquisition and maintenance of necessary skills. Also, it raises sustainability concerns, given the time-bound nature of its implementation [79].

The DOCFR exceeded the maximum acceptable level, suggesting poor quality of EmONC. Similar findings were reported in DRC and other SSA countries [50, 62, 63, 80–82]. However, given the observation that all direct obstetric deaths occurred only in the referral facilities, this indicator might also be reflective of the state of referral systems in the study HZs. Inadequate means of transportation and communication to link referring and receiving facilities may have contributed to the delayed arrival of unannounced referrals of women with MDOC at higher levels of EmONC, further compromising the prognosis. Maternal mortality audits in resource-constrained settings have pointed to a number of barriers to timely access to EmONC, including inappropriate and ineffective referral, inefficient ambulance services, cost of transportation, and long waiting time before care is received at higher-level facilities [83, 84].

The population-based CS rate was the only process indicator found to be within an acceptable range in 2 of our study HZs. This is in contrast with findings from an EmONC study in Lubumbashi, the second largest city in DRC, barely affected by the 2006–2013 conflict episodes, that revealed an unmet need for CS deliveries [63]. These CS rates in the study HZs are rather encouraging, reflecting the contribution of humanitarian actors and NFPOs to the provision of public maternal and newborn health care services in the region. Even in the HZ of Karisimbi where none of the facilities qualified as functioning EmONC facility, the population-based CS rate was within the limits set by the WHO.

Since only a little over one-sixth of deliveries occurred in functioning EmONC and given the shortcomings in referral capabilities and unmet minimum acceptable levels of use and quality, one can surmise that most pregnancy-related complications had likely received substandard or delayed care. One systematic review found an inverse correlation between the met need for EmONC and maternal mortality ratio [85], highlighting the impact of better access to quality EmONC on reducing preventable mortality, a key element in post-conflict recovery. A study in Nigeria identified factors contributing to maternal mortality, including a dysfunctional referral system and limited intensive care capabilities causing delays in providing EmONC [84].

This study has some limitations. First, only three out of the 34 HZs in the North-Kivu Province were included in a purposive manner and, therefore, our findings and analyses cannot be generalized to the whole Province. The EmONC status in HZs located further to the 2006–2013 conflict events might be different. Second, profit-run health facilities were not selected, which very likely underestimated the provision and capacity of EmONC as well as facility deliveries in the 3 HZs. Our findings can also not be extrapolated to these facilities. Third, we did not assess staff knowledge of EmONC procedures and standards of care, leaving gaps in the actual ability to provide quality care in the study HZs and calling for further research. Fourth, we collected data in 2017 and this might raise concerns as to the current pertinence of our findings. Nevertheless, recent qualitative findings on health services for women, children, and adolescents in North Kivu, highlighting human resource and logistic barriers, might suggest that this study remains relevant [86].

## Conclusion

This study gives for the first time a quantitative assessment of the post-conflict status of EmONC in the North-Kivu Province in eastern DRC. By identifying the gaps in the availability, use and quality of EmONC in the study HZs, it raises the prospect for evidence-based policies and programming, as well as coordinated EmONC interventions, which should encourage local and external stakeholders to work together in improving maternal and neonatal health in the province as part of the post-conflict recovery efforts. Despite the fact that most process/performance indicators didn’t meet the WHO standards, EmONC services were provided to a degree that appeared to be more advantageous for urban populations and more comprehensive in public referral facilities, especially where the NFPO or humanitarian partners were involved. Special attention to basic EmONC and referral linkage among public health facilities is required, particularly in non-urban HZs, with an emphasis on strengthening human resources, equipment, supply chains, and referral capacity, on the one hand, and on tackling residual insecurity that might hinder 24/7 staff availability, on the other hand. In view of the marked inadequacy between the number of HCs (designated bEmONC points) and the provision of basic signal functions across the study HZs, initial efforts to improve access to quality EmONC could be directed towards upgrading the capacities of a few HCs (i.e., through supplying and equipping them appropriately, as well as through in-service training programmes), starting with those located in non-urban HZs. There is also the need to review the existing policies with respect to restrictions on the provision of EmONC signal functions in primary health care centres, particularly on AVD and neonatal resuscitation, provided appropriate planning and support. This study was conducted before the 2018 Ebola outbreak in North Kivu. The Ebola outbreak disrupted the provision of, and access to, essential health services, including EmONC [87]. Thus, our data can form a baseline for a follow-up survey to update and evaluate the status of EmONC service provision in these HZs.

## Data Availability

The minimal dataset necessary to interpret and build upon our findings is included in this article. Further dataset inquiry can be submitted to the corresponding author upon reasonable request.

## Abbreviations

EmONC: emergency obstetric and newborn care
NKP: North Kivu Province
DRC: Democratic Republic of the Congo
HZ: Health zone
SSA: Sub-Saharan Africa;
IAWG: The Inter-Agency Working Group for Reproductive Health
SRH: sexual and reproductive health
UN: United Nations
MRP: Manual removal of placenta
RRP: Removal of retained products of conception
AVD: Assisted vaginal delivery
NR: Neonatal resuscitation
CS: Caesarean section
CNDP: Congrès National pour la Défense du Peuple
M23: Mouvement du 23 Mars
CBHZ: Central Bureau of the Health Zone
HC: Health centre
RHC: Referral health centre
RH: Referral hospital
MOH: Minister of Health
AMDD: Averting Maternal Death and Disability
WHO: World Health Organization
UN: United Nations
UNICEF: United Nations’ Children Fund
UNFPA: United Nations Population Funds
CMO: Chief Medical Officer
PI: Principal investigator
NFPO: non-for-profit organisations
MSF: Médecins Sans Frontières
USD: United States Dollars
MDOC: Major direct obstetric complications
DOCFR: Direct obstetric case facility rate
INMR: Intrapartum and very early neonatal death rate
IQR: Interquartile Range
SPE: Severe pre−/eclampsia
